# Hypozincemia in COVID-19 Patients Correlates With Stronger Antibody Response

**DOI:** 10.3389/fimmu.2021.785599

**Published:** 2022-01-04

**Authors:** Wenye Xu, Yingzhi Liu, Xuan Zou, Huanle Luo, Weihua Wu, Junjie Xia, Matthew T. V. Chan, Shisong Fang, Yuelong Shu, William K. K. Wu, Lin Zhang

**Affiliations:** ^1^Department of Medicine and Therapeutics, The Chinese University of Hong Kong, Hong Kong, Hong Kong SAR, China; ^2^Department of Anaesthesia and Intensive Care and Peter Hung Pain Research Institute, The Chinese University of Hong Kong, Hong Kong, Hong Kong SAR, China; ^3^Shenzhen Center for Disease Control and Prevention, Shenzhen, China; ^4^School of Public Health (Shenzhen), Sun Yet-Sen University, Shenzhen, China; ^5^Key Laboratory of Tropical Disease Control, Ministry of Education, Sun Yat-sen University, Guangzhou, China; ^6^State Key Laboratory of Digestive Diseases, Li Ka Shing Institute of Health Sciences, Prince of Wales Hospital, The Chinese University of Hong Kong, Hong Kong, Hong Kong SAR, China

**Keywords:** zinc, RBD, antibody, SARS – CoV – 2, COVID - 19

## Abstract

Zinc ion as an enzyme cofactor exhibits antiviral and anti-inflammatory activity during infection, but circulating zinc ion level during Severe Acute Respiratory Syndrome Coronavirus 2 (SARS-CoV-2) infection is unclear. This study aimed to evaluate serum zinc ion level in Coronavirus Disease 2019 (COVID-19) patients and healthy subjects, as well as its correlation with antibodies against SARS-CoV-2. 114 COVID-19 patients and 48 healthy subjects (38 healthy volunteers and 10 close contacts of patients with COVID-19) were included. Zinc ion concentration and levels of antibodies against SARS-CoV-2 Spike 1 + Spike 2 proteins, nucleocapsid protein, and receptor-binding domain in serum were measured. Results showed that the concentration of zinc ion in serum from COVID-19 patients [median: 6.4 nmol/mL (IQR 1.5 – 12.0 nmol/mL)] were significantly lower than that from the healthy subjects [median: 15.0 nmol/mL (IQR 11.9 – 18.8 nmol/mL)] (*p* < 0.001) and the difference remained significant after age stratification (*p* < 0.001) or when the patients were at the recovery stage (*p* < 0.001). Furthermore, COVID-19 patients with more severe hypozincemia showed higher levels of IgG against the receptor-binding domain of SARS-CoV-2 spike protein. Further studies to confirm the effect of zinc supplementation on improving the outcomes of COVID-19, including antibody response against SARS-CoV-2, are warranted.

## Introduction

Therapeutic options for Coronavirus Disease 2019 (COVID-19) are currently limited. Zinc as an enzyme cofactor has been shown to preserve respiratory epithelium, prevent pathogen entry and modulate the antiviral and inflammatory responses (1). Recently, the MATH+ protocol formulated by the Frontline COVID-19 Critical Care Alliance incorporated zinc supplementation as an optional co-intervention for all hospitalized COVID-19 patients (2). Anecdotal evidence also suggests treatment with high-dose zinc salt might improve outcomes of COVID-19 patients (3). However, the role of zinc levels in modulating on the immune response against Severe Acute Respiratory Syndrome Coronavirus 2 (SARS-CoV-2) in COVID-19 patients is so far unclear.

Herein, we conducted a retrospective study to analyze the serum zinc ion concentration in 162 blood specimens collected from 114 COVID-19 patients and 48 healthy subjects and its correlation with the immune response profile.

## Methods

### Patient Recruitment

We included 114 COVID-19 patients that were tested positive for SARS-CoV-2 in throat swabs, based on real-time reverse transcription (RT)-polymerase chain reaction (PCR) according to a standard protocol (4). Among them, 50 patients underwent serum antibody level measurement [23 samples collected from the acute phase (Day 7 to day 21) and 27 samples from the late phase (after day 21)]. We also included 48 subjects that were healthy volunteers (n = 38) or close contacts of COVID-19 patients (*n* = 10). These control subjects were asymptomatic and tested negative for SARS-CoV-2 by IgG, IgM, and real-time reverse transcription-PCR. Serum samples from COVID-19 patients were collected at the median time from symptom onset of 12 days (IQR 7-19.25 days). This work was done to support an ongoing public health response and data collection is part of the continuing public health investigation of an emerging outbreak and therefore the individual informed consent was waived (5). The study was approved by the ethics committee of the Shenzhen Center for Disease Control and Prevention (CDC).

### Clinical Data Collection

We collected the patient details and clinical outcomes through reviewing patient medical records. All data were recorded on a specifically designed data collection form. To ensure the data accuracy, two researchers independently reviewed the clinical notes and laboratory results. Disagreements were resolved by consensus.

### Determination of Amount of Zinc Ion in Serum

Blood samples were collected during the hospital stay and after discharge as clinically indicated. Whole blood samples were allowed to set at 4°C for 60 minutes. After centrifuging at 1,500× g for 10 minutes, the supernatant was collected and stored at -30°C until assay. Serum concentrations of zinc ion were measured using a commercially available zinc ion quantification kit (ab102507; Abcam) according to the manufacturer’s instructions.

### SARS-CoV-2 Antibody Measurements

Enzyme-linked immunosorbent assays (ELISA) from Sino Biological Inc company were applied to detect SARS-CoV-2 antibodies isotypes including IgA, IgG, and IgM against the spike protein (S), the nucleocapsid protein (NP), and the receptor binding domain (RBD) in serum. 96-well EIA plates were coated with S1+S2, NP or RBD separately overnight at 4°C following by blockade with 200 μl/well of 2% Bovine Serum Albumin (Sigma-Aldrich, USA) in phosphate-buffered saline solution with 0.05% Tween™ 20 (PBST) overnight at 4°C. Serum samples were diluted in ten-fold dilutions with blocking buffer and 100 μl/well of samples were then added to the plates and incubated for two hours at room temperature (RT). Then, plates were washed three times with PBST and added with HRP-labeled goat anti-human IgA, IgG, IgM (Abcam, UK) or horseradish peroxidase-labelled mouse anti-human IgG1-4 (Southern Biotech, USA). After incubation at RT for one hour, plates were incubated with 3,3′,5,5′-tetramethylbenzidine (Solarbio, China) for 25 min at RT after washing six times. The reaction was stopped with the ELISA stop solution (Solarbio, China). Then the OD_450_ was read on BioTek (Synergy HTX, USA). Serum sample FS B26 with strong neutralization activity in micro-neutralization assay kindly provided by the Guangdong Provincial CDC diluted in ten-fold dilutions was set as a positive control on every ELISA plate to normalize all the detected values on different plates (6).

### Statistics

Data were expressed as median and interquartile range (IQR) or range and counts with percentages, as appropriate. Differences in serum zinc ion levels between COVID-19 patients and healthy subjects were compared using Wilcoxon test. Spearman’s rank-sum correlation coefficient was used to determine the correlation between serum zinc ion concentrations and antibody levels against SARS-CoV-2 S1+S2, NP and RBD.

## Results

**Figure 1:**
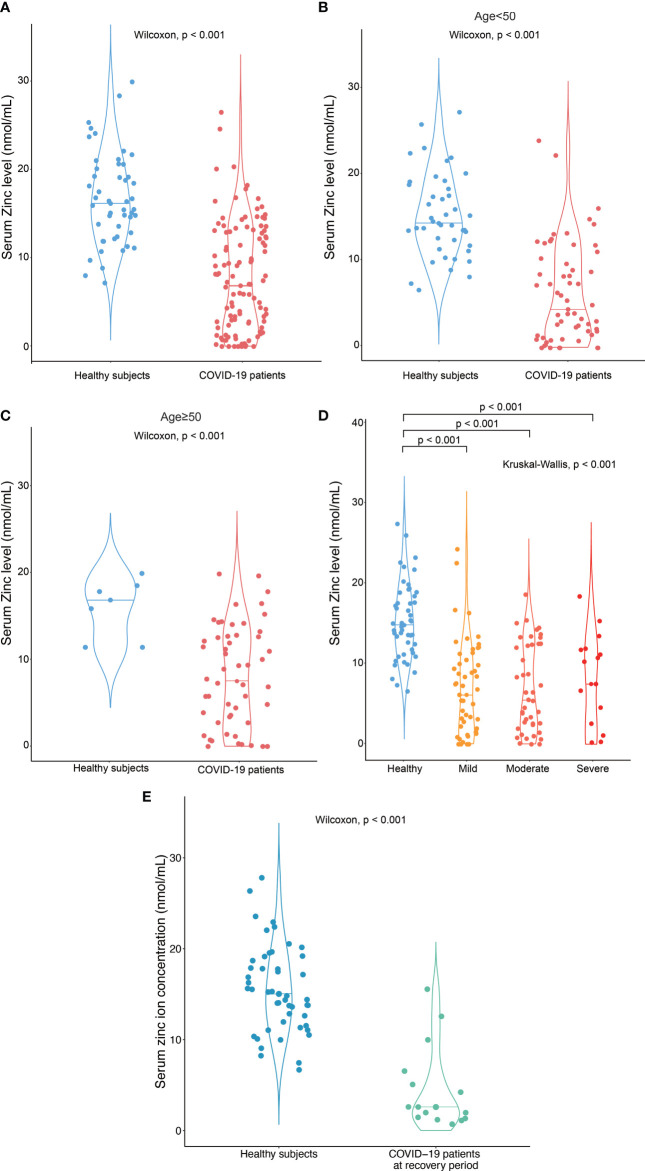
Hypozincemia in patients with COVID-19. **(A)** Serum zinc ion concentrations in patients with COVID-19 (n = 114) were significantly lower than that in healthy subjects (n = 48). (B-D) The difference in serum zinc ion concentrations remained significant when the subjects were stratified by age or disease severity – **(B)** subjects < 50 years old; **(C)** subjects ≥ 50 years old; **(D)** COVID-19 patients with different severity classified by their need for oxygen therapy and ventilatory support; significantly different between the indicated groups by Wilcoxon test; **(E)** Serum zinc ion concentration in patients with COVID-19 at recovery stage (after discharge to the hospital) (n = 15) were significantly lower than that in healthy subjects (n = 48).

**Figure 2:**
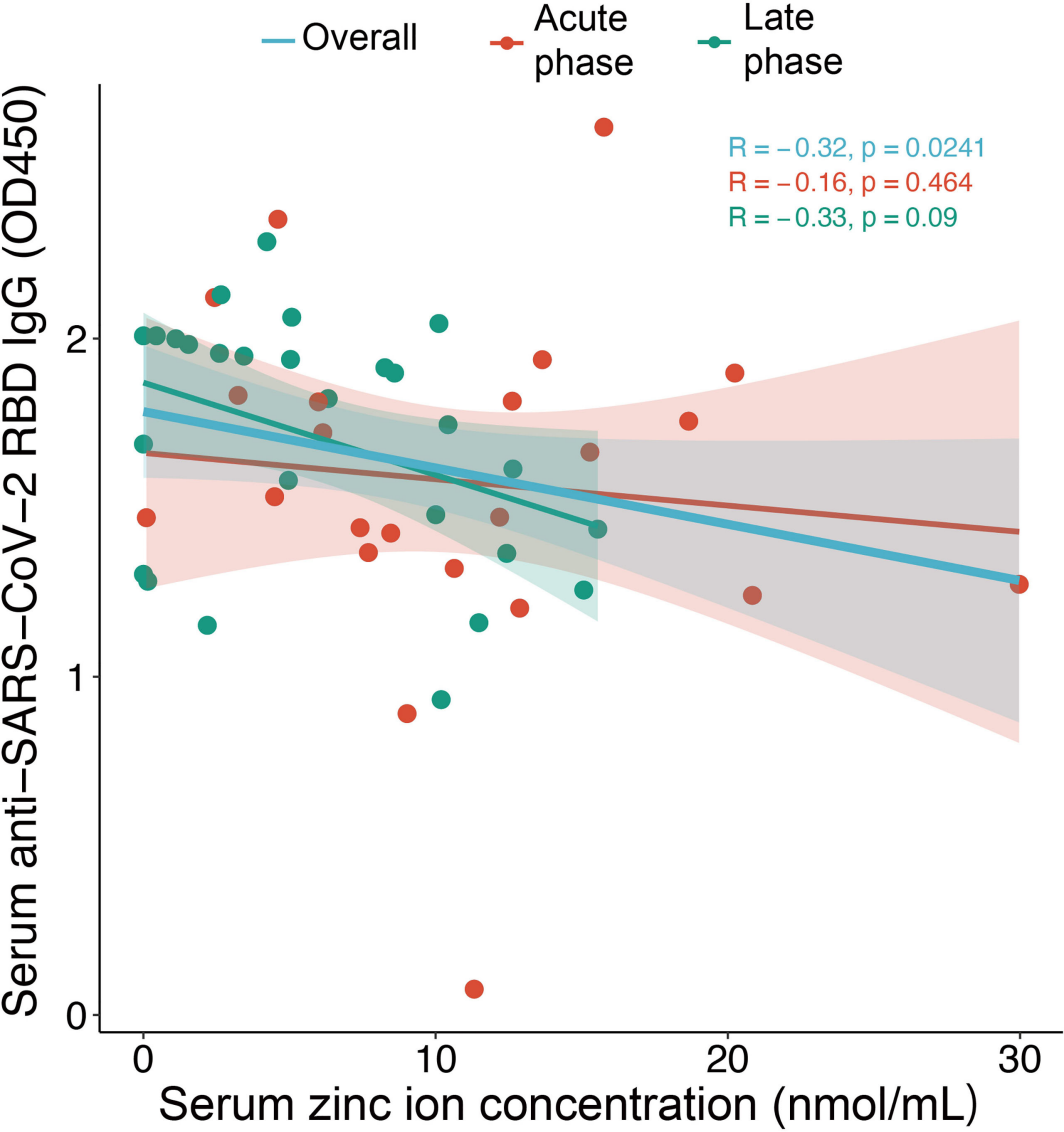
Correlation between serum zinc ion concentrations and anti-SARS-CoV-2 RBD IgG level for samples collected in the acute phase (day 7-21) (n = 23) and the late phase (after day 21) (n=27) from COVID-19 patients. The Spearman’s rank-sum correlation coefficient and significance are shown. Shading represents 95% confidence intervals (CIs).

Among 114 COVID-19 patients, 54 subjects (47%) were male and the median age was 49.5 (IQR:35 – 61) years. For the healthy subjects, consisted of 38 healthy volunteers and 10 close contacts of patients with COVID-19 in this cohort, 25 subjects were male and the median age was 39 (IQR:30-47.25) years. The serum zinc ion concentration in COVID-19 patients [median: 6.4 nmol/mL (IQR 1.5 – 12.0 nmol/mL)] was significantly lower than that in the healthy subjects [median: 15.0 nmol/mL (IQR 11.9 – 18.8 nmol/mL)] (*p* < 0.001) (**Figure 1A**). This difference remained significant after age stratification (*p* < 0.001) (**Figures 1B, C****)**. There was, however, no correlation between zinc ion concentration and disease severity (**Figure 1D**). Furthermore, the zinc ion concentration of COVID-19 patients at the stage of recovery was still significantly lower than that of the healthy subjects (*p* < 0.001) (**Figure 1E**). Most of the determined antibodies against SARS-CoV-2 exhibited negative correlations with zinc ion level (**Supplementary Figure 1**), yet only the anti-SARS-CoV-2 receptor RBD IgG reached statistical significance (**Figure 2**).

## Discussion

Our study shows that patients with COVID-19 had a significantly lower serum zinc ion concentration than healthy individuals. Despite the lack of evidence on the direct effect of zinc ion on SARS-CoV-2, its antiviral effects have been demonstrated in various viral infections (7). As an antiviral nutrient, zinc ion was reported to be able to inhibit replication of rhinoviruses, respiratory syncytial virus (8, 9), picornavirus (10), and the RNA polymerase activity of SARS-associated coronavirus and equine arteritis virus (11). Similar to our finding, hypozincemia was reported in paediatric patients with severe pneumonia or Dengue viral infection (12, 13). Given that serum zinc accounts for only 0.1% of the body’s total zinc amount (14), it is likely that an altered zinc distribution between the intracellular pool and the circulation during infection might explain the observed hypozincemia. In this connection, interleukin-6-mediated induction of Zip14/SLC39A14 is known to mediate the uptake of zinc into the liver during acute inflammation and infection, which is believed to be host defensive (15). However, the exact function of altered zinc distribution remains uncertain.

The most notable finding of the present study is that COVID-19 patients with lower serum zinc ion concentration exhibited stronger antibody response against SARS-CoV-2, particularly, the RBD. The SARS-CoV-2 RBD was reported to have strong binding affinity to the host angiotensin-converting enzyme 2 (ACE2) which mediates the viral entry (16, 17). One possibility is that patients with stronger antibody response had higher extent of B cell activation and expansion. In this connection, the uptake of transferrin-bound zinc has been shown to be stimulated in human lymphocytes upon activation (18). Nevertheless, the effect of zinc ion on antibody response, especially in the context of vaccination, is controversial. It was demonstrated that supplementation with zinc is associated with improvement of seroconversion to vibriocidal antibody in 2-5-year-old children receiving cholera vaccine (19) and is able to boost higher levels of anti-HBs titers in infant receiving hepatitis B vaccination (20). However, previous studies also showed that zinc supplementation could not promote immunogenicity of rotavirus vaccine (21) or enhance antibody response to multivalent influenza vaccine in hemodialysis patients (22).

A second remarkable finding of this study is that hypozincemia among COVID-19 patients did not resolve even they were at the stage of recovery, suggesting that COVID-19-associated hypozincemia is not transient. This is reminiscent of the emerging “long COVID”, in which symptoms such as fatigue, dyspnea and cardiac abnormalities can persist long after the acute phase of infection (23). The pathogenic mechanism underlying the persistent hypozincemia and its relationship with other abnormalities observed in patients with long COVID require further investigation.

Finally, another key finding is that all COVID-19 patients having mild, moderate and severe disease manifestation had a similar extent of hypozincemia. This is inconsistent with two previous studies revealing the association between low serum zinc ion concentrations and severe COVID-19 (24, 25). Nevertheless, it is worthwhile to note that the post-infectious serum concentration of zinc ion depends on both the premorbid zinc level and the host hypozincemic response during infection. Therefore, the prevalence of zinc deficiency in different geographical regions could affect how the finding is interpreted.

In summary, COVID-19 patients had significantly lower serum zinc ion concentrations than healthy individuals. A higher extent of hypozincemia was also associated with a stronger antibody response against SARS-CoV-2 RBD. Our study has several limitations. First, we do not have comparable numbers of healthy control subjects and COVID-19 patients. Second, the mechanism underlying the association between low zinc ion concentrations and high anti-SARS-CoV2 RBD IgG levels remains uncertain.

We cannot rule out the possibility that both were commonly caused by higher viral load. Last but not least, the promulgation that zinc supplementation could be of benefit for the treatment of COVID-19 awaits confirmation by randomized clinical trials.

## Data Availability Statement

The raw data supporting the conclusions of this article will be made available by the authors, without undue reservation.

## Ethics Statement

The studies involving human participants were reviewed and approved by ethics committee of the Shenzhen Center for Disease Control and Prevention. Written informed consent for participation was not required for this study in accordance with the national legislation and the institutional requirements.

## Author Contributions

LZ, WKKW and MTVC made substantial contribution to the conception, design of the work and interpretation of data. WYX, YZL and HLL carried out serology testing and analysis. LZ, WKKW, WYX, YZL and MTVC wrote the manuscript with input from all co-authors. SSF, XZ, and YLS contributed on the samples collection and manuscript revision. All authors contributed to the article and approved the submitted version.

## Conflict of Interest

The authors declare that the research was conducted in the absence of any commercial or financial relationships that could be construed as a potential conflict of interest.

## Publisher’s Note

All claims expressed in this article are solely those of the authors and do not necessarily represent those of their affiliated organizations, or those of the publisher, the editors and the reviewers. Any product that may be evaluated in this article, or claim that may be made by its manufacturer, is not guaranteed or endorsed by the publisher.

## Supplementary Material

Supplementary Figure 1 Correlations between serum zinc ion concentrations and levels of antibodies against SARS-CoV-2 proteins. Correlations between serum zinc ion levels and anti-SARS-CoV-2 NP **(A, D)**, RBD **(B, E)**, S1+S2 protein **(C, F)** IgA, IgM and IgG, IgG1, IgG2, IgG3, IgG4 levels as well as neutralizing antibody **(G)** are shown. Samples were collected in the acute phase (day 7-21) (n=23) and the late phase (after day 21) (n=27) from COVID-19 patients. The Spearman’s rank-sum correlation coefficient and significance are shown.
