# Supplementary figures and images for: Hypozincemia in COVID-19 Patients Correlates With Stronger Antibody Response
Source: Front Immunol. 2022 Jan 4;12:785599. doi: 10.3389/fimmu.2021.785599 (PMC8763690; doi:10.3389/fimmu.2021.785599)

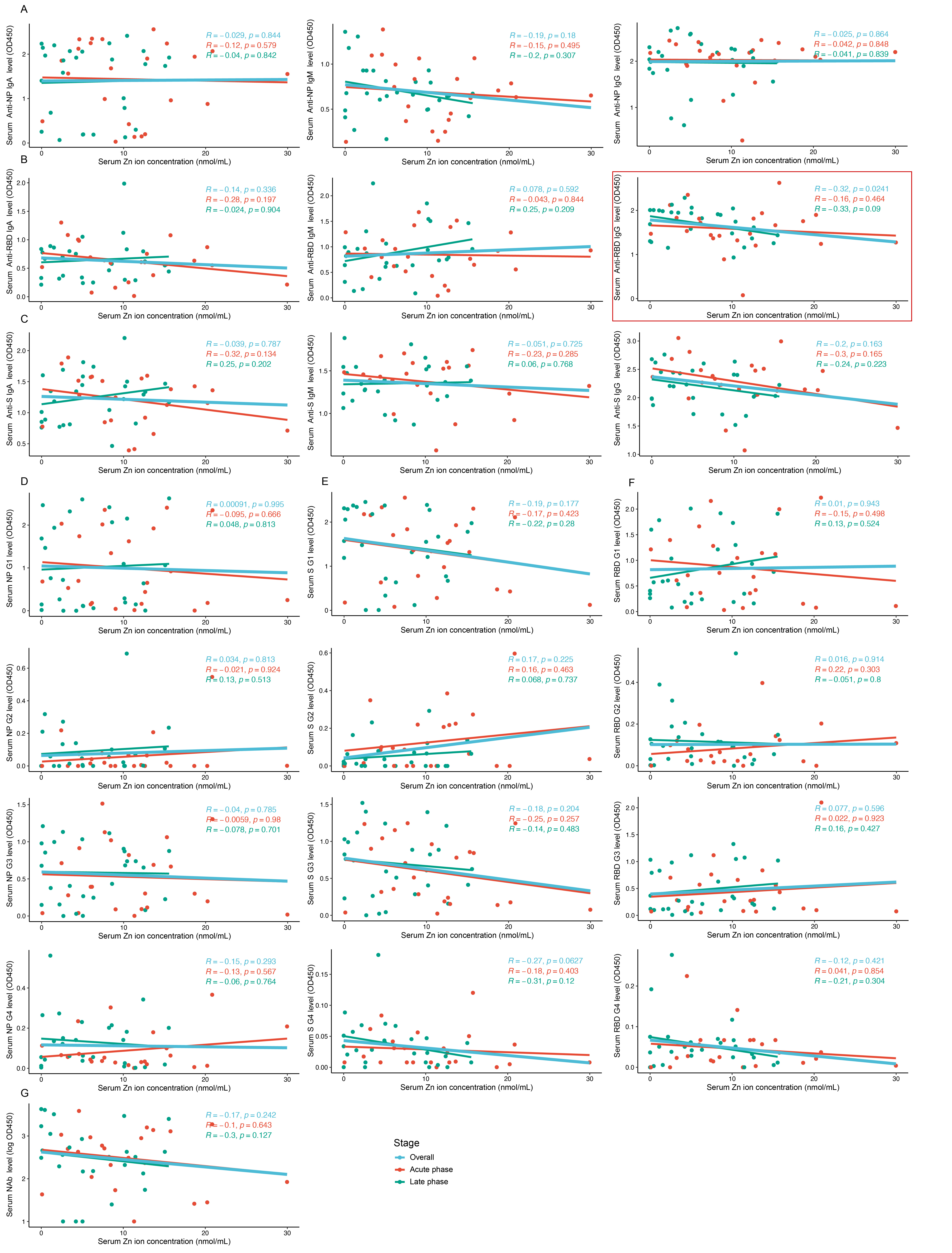

Supplement: Supplementary Figure 1 — Correlations between serum zinc ion concentrations and levels of antibodies against SARS-CoV-2 proteins. Correlations between serum zinc ion levels and anti-SARS-CoV-2 NP (A, D), RBD (B, E), S1+S2 protein (C, F) IgA, IgM and IgG, IgG1, IgG2, IgG3, IgG4 levels as well as neutralizing antibody (G) are shown. Samples were collected in the acute phase (day 7-21) (n=23) and the late phase (after day 21) (n=27) from COVID-19 patients. The Spearman’s rank-sum correlation coefficient and significance are shown. [file Image_1.jpeg]
